# The Cervicovaginal Mucus Barrier

**DOI:** 10.3390/ijms21218266

**Published:** 2020-11-04

**Authors:** Guillaume Lacroix, Valérie Gouyer, Frédéric Gottrand, Jean-Luc Desseyn

**Affiliations:** Inserm U1286, Infinite, Univ. Lille, CHU Lille, F-59000 Lille, France; guillaume-p.lacroix@inserm.fr (G.L.); valerie.gouyer@inserm.fr (V.G.); frederic.gottrand@chru-lille.fr (F.G.)

**Keywords:** bacterial vaginosis, cervical mucus plug, cervix, microbiota, mucin, mouse model

## Abstract

Preterm births are a global health priority that affects 15 million babies every year worldwide. There are no effective prognostic and therapeutic strategies relating to preterm delivery, but uterine infections appear to be a major cause. The vaginal epithelium is covered by the cervicovaginal mucus, which is essential to health because of its direct involvement in reproduction and functions as a selective barrier by sheltering the beneficial lactobacilli while helping to clear pathogens. During pregnancy, the cervical canal is sealed with a cervical mucus plug that prevents the vaginal flora from ascending toward the uterine compartment, which protects the fetus from pathogens. Abnormalities of the cervical mucus plug and bacterial vaginosis are associated with a higher risk of preterm delivery. This review addresses the current understanding of the cervicovaginal mucus and the cervical mucus plug and their interactions with the microbial communities in both the physiological state and bacterial vaginosis, with a focus on gel-forming mucins. We also review the current state of knowledge of gel-forming mucins contained in mouse cervicovaginal mucus and the mouse models used to study bacterial vaginosis.

## 1. Introduction

Mucus is a complex viscoelastic gel that forms on the surface of the secretory epithelium and acts as the first line of defense against harmful agents from the outside environment [1]. Mucus of the woman’s genital tract (WGT) plays an essential role in many biological functions. It moisturizes the genital tract mucosa, lubricates the lower genital tract during sexual intercourse, and allows or stops sperm cells from ascending toward the ovule depending on the phase of the menstrual cycle [2,3].

**Figure 1:**
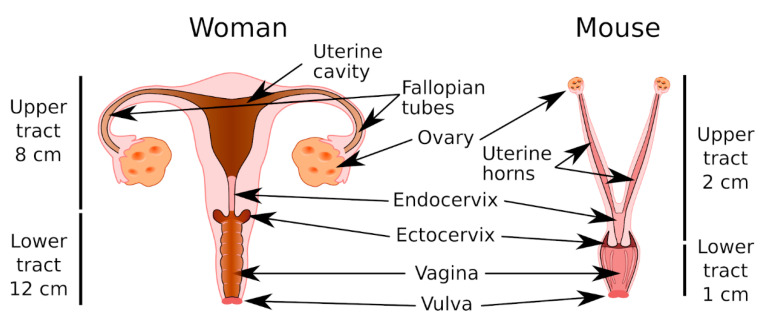
Women have a large pear-shaped uterine cavity. The transition between the uterus and vagina occurs in the cervical canal between the mucus-secreting endocervical epithelium and the stratified squamous epithelium of the ectocervix. The vagina is lined by a squamous and muscular epithelium that is not keratinized. The mouse uterus is bicornuate. As for the woman, the lower genital tract in the mouse contains the cervix, which includes the endocervix and the ectocervix, and opens into the vaginal cavity. The mouse vaginal epithelium is keratinized.

The endocervical epithelium is the main source of mucus secreted in the WGT (Figure 1). Endocervical mucus migrates along the cervical canal to the vaginal cavity, where it mixes with numerous compounds secreted by the host, cellular debris, and vaginal microbiota to form cervicovaginal mucus (CVM), which is an important ecological niche that houses the vaginal flora, which play an essential role in maintaining the vaginal mucosal barrier [4]. The vaginal flora is dominated by lactobacilli, and depletion of the vaginal lactobacilli, a hallmark of bacterial vaginosis, promotes the proliferation of opportunistic or sexually transmitted pathogens, and can lead to vaginal infection or vaginosis. Bacterial vaginosis is particularly serious in pregnant women because it is often associated with premature birth [5]. During pregnancy, a cervical mucus plug (CMP) is formed in the cervical canal that isolates the nearly sterile uterus from the vaginal compartment and thereby protects the fetus against vaginal pathogens. Therefore, the immune and viscoelastic properties of CMP are essential for preventing bacterial ascension to the uterus [6].

The purpose of this review is to provide a comprehensive overview of CVM and the CMP and to describe the relationships between the mucus of the WGT and the microbial communities in both the physiological state and bacterial vaginosis. We also review the limited current knowledge obtained from animal models with a focus on the laboratory mouse, which is the most common animal used for exploring in vivo the mucus gel, gel-forming mucins (GFMs), and preterm birth induced by experimental bacterial vaginosis.

## 2. Cervicovaginal Mucus

### 2.1. Composition

#### 2.1.1. Gel-Forming Mucins

Water is the main component of mucus (>95% by weight), and GFMs represent more than 80% of the mucus organic fraction [7]. Gel-forming mucins are *N*-glycosylated and heavily *O*-glycosylated proteins that polymerize by disulfide bonds through their amino- and carboxy-terminal regions to form long polymers that are then secreted into the lumen by serous and goblet cells, and by submucosal glands. Once secreted, GFMs form a heterogeneous protein network and are the main drivers of the viscoelastic properties of the mucus [1]. The five human GFMs are conserved in mammals and are all made of a large central part enriched in Ser/Thr/Pro, which is flanked by conserved globular regions enriched in cysteine residues, which is also found in the von Willebrand factors [8]. The four GFMs, MUC2, MUC5AC, MUC5B, and MUC6, have been found in the WGT (Table 1). To date, no studies have found MUC19 in the WGT.

A hallmark of the three GFMs, MUC2, MUC5AC, and MUC5B (and their animal orthologs), is the presence in multiple copies of a hydrophobic domain enriched with cysteine residues called the mucin CYS domain. This domain is highly conserved and always contains the Trp–Xaa–Xaa–Trp signature, which is believed to be *C*-mannosylated and to establish noncovalent bridges between GFMs [12,13,14,15]. For each human tissue, as for the WGT, there is usually not a single GFM expressed but at least two GFMs with always at least one GFM containing the CYS domain [1].

**Figure 2:**
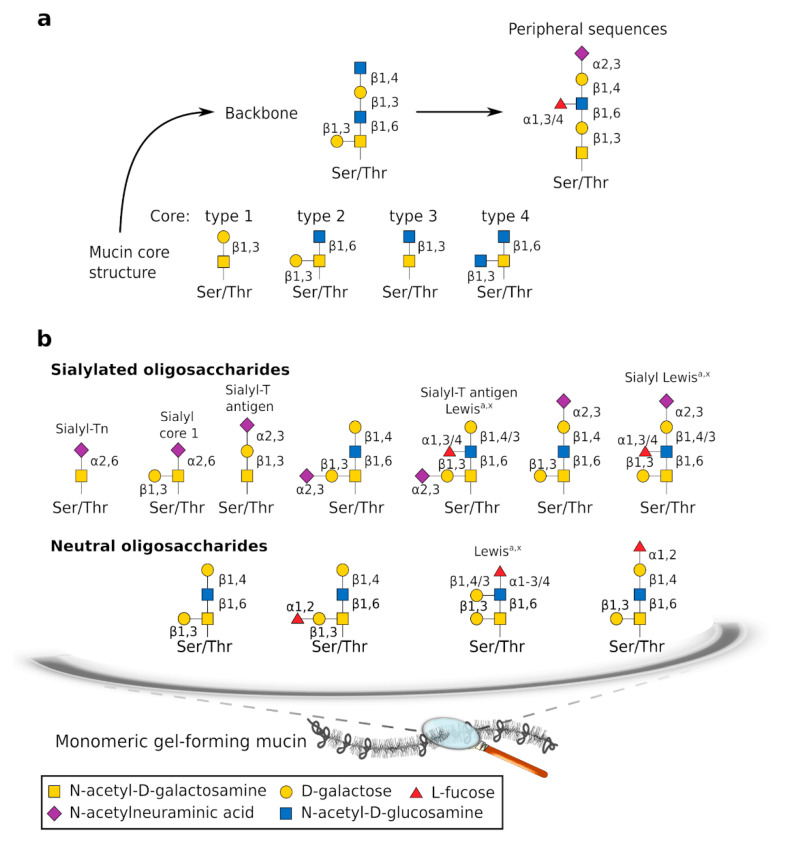
Oligosaccharide chains of human GFMs. (**a**) Theoretical *O*-glycosylation. The backbone is made of repeated units, which are not represented. Among the eight known types of mucin core structure, cores 1–4 are the most common. (**b**) Examples of the main GFM glycans of cervicovaginal mucus (CVM), which are mainly sialylated and fucosylated. More neutral oligosaccharides than sialylated oligosaccharides are detected at midcycle than at pre- or post-ovulation. The sugar code letter and *O*-glycan structure are from Kaltner et al. [17].

The CYS domains are found irregularly interspaced within the central part of GFMs where they are surrounded by large regions enriched with hydroxyamino acids and proline (S/T/P regions) that are common to all mucins and organized in tandem repeats, at least in humans. Regions rich in S/T/P are substituted with large *O*-glycan chains [16]. Glycans constitute the main component of mature GFMs and confer a hydrophilic characteristic to GFMs. Glycan chains are synthesized sequentially by specific enzymes. All *O*-glycosylation chains have a core, some have a backbone made of repeat glycans, and most have peripheral additions that carry sulfate and sialic acid residues, which give a net negative charge (Figure 2a). Eight core structures have been determined and four are widespread. For a more comprehensive review of mucin glycosylation, we refer interested readers to the References [17,18,19].

Once GFMs are secreted, the mucin-associated glycans bind water through hydrogen bonds, which induces GFM hydration and the gelation process. The GFM network is also modulated by weaker forces such as hydrophobic and electrostatic interactions related to the pH, ionic strength, and the oxidation–reduction potential of the microenvironment [1].

#### 2.1.2. Other Components

Cervicovaginal mucus is a complex hydrogel that contains, in addition to water and GFMs, a mixture of proteins and molecules that are secreted by the host cells and vaginal flora. Cervicovaginal mucus comprises endometrial fluids containing exfoliated epithelial cells, nucleic acids released by neutrophils or from dead cells, lipids, and mucin-associated fatty acids, electrolytes, such as sodium and potassium chloride, and numerous proteins that function in immune defense.

Large amounts of trefoil factor family (TFF) peptides are found in the CVM proteome. The three human TFFs—TFF1, TFF2, and TFF3—are cosecreted with GFMs by mucin-producing cells [20]. The TFF expression varies during the menstrual cycle and increases in the ovulatory phase. The median concentrations of TFF1, TFF2, and TFF3 normalized to total protein concentration in cervical mucus are 3.2, 0.88, and 530 nmol/g protein during ovulation, respectively. The highest concentration of TFF3 found in human fluids was in the cervical mucus, which suggests an important role of TFF3 in the WGT [21]. The biological role of TFFs in the WGT remains unclear, but TFF binding to GFMs is thought to play the role of a noncovalent cross-linking between GFMs, which modulates the viscoelastic properties of the mucus hydrogel [22,23].

Among the molecules involved in host immune defense, proteins secreted as part of adaptive immunity, such as immunoglobulins (Ig), play a crucial role. For example, high concentrations of secretory IgA, IgG, and IgM strengthen the mucosal barrier against infection in CVM [24,25]. The IgG may interact directly with mucins through multiple low-affinity bonds and may, if present in sufficient quantities, effectively neutralize virions of the human immunodeficiency virus (HIV) in CVM [26].

Cervicovaginal mucus is enriched with immune cells, such as neutrophils, macrophages, and monocytes, which secrete small antimicrobial peptides (AMPs, <100 amino acids) such as α-defensins, β-defensins, and lysozyme [27]. Epithelial cells also contribute to the arsenal of AMPs by producing lactoferrin, cathelicidin, calprotectin, and trappin-2/elafin [28]. In addition to their antimicrobial activity, AMPs exhibit immunomodulatory properties and participate in epithelial homeostasis. Together, all secreted immune proteins and GFMs provide effective immune protection against pathogens and sexually transmitted infection.

### 2.2. Hormonal Regulation

**Figure 3:**
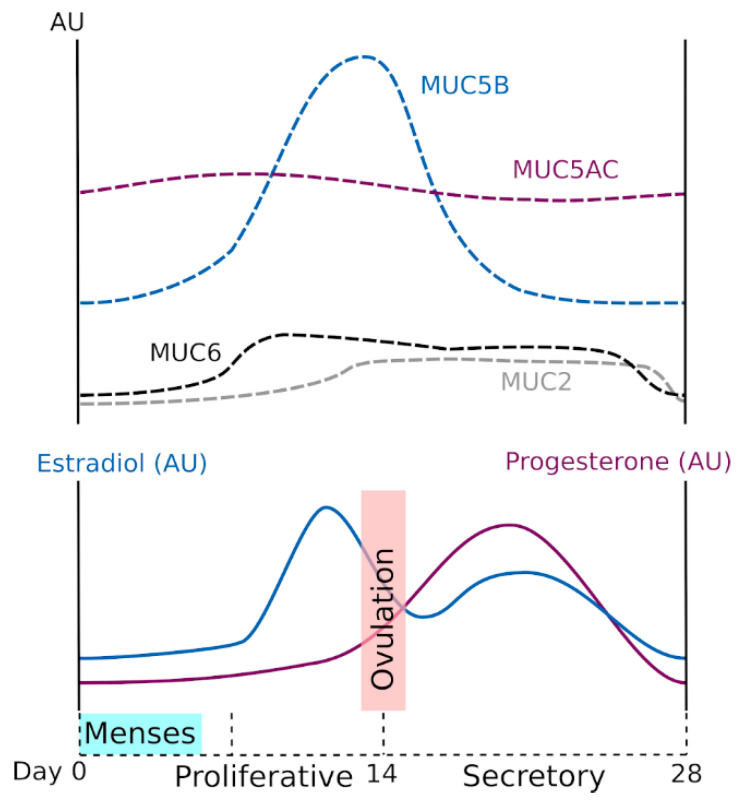
Hormonal regulation of GFMs in the woman’s genital tract. MUC5B was the main GFM in the woman’s genital tract, and its expression peaked at midcycle. MUC5AC was highly expressed and appeared to have little variation through the menstrual cycle. MUC6 and MUC2 were detected in small amounts. MUC6 was observed during the proliferative and secretory phases, and MUC2 during the secretory phase. AU, arbitrary units.

The WGT experiences significant changes in anatomy and physiology over the life course, including menstruation, pregnancy, parturition, and menopause [29]. These different physiological states are highly regulated by sex hormones. Modification of the genital tract epithelia and mucus support each new biological function, and CVM and GFM expression is highly dependent on both natural and artificial hormonal changes [30,31,32]. Proteomic analysis has identified specific proteins in the cervical mucus of the preovulatory, ovulatory, and postovulatory phases [33]. The composition and viscoelasticity of CVM change during the menstrual cycle. Variations in CVM viscoelasticity allow the identification of the fertile window, which can be used to help promote pregnancy because, near the time of ovulation, the cervical mucus becomes waterier and more permeable to spermatozoa [30,34]. These changes appear to be influenced mainly by variations in mucin concentration and mucin-associated *O*-glycans because cervical mucus production increases in midcycle and is accompanied by modification of mucin glycosylation [35,36,37]. At midcycle, GFMs contain more neutral oligosaccharides than in the pre- or postovulatory phases, which is probably important for fecundity [31,38] (Figure 2b). Gel-forming mucin expression has been examined using a range of molecular techniques at the messenger RNA (in situ hybridization, Northern blot, reverse transcription polymerase chain reaction) and protein levels (enzyme-linked immunosorbent assay, Western blot analysis, immunohistochemistry, mass spectrometry). Both MUC6 and MUC2 expression appear to be low but to vary along the menstrual cycle. In addition, MUC5AC and MUC5B are the two main GFMs, and expression is probably higher for MUC5B than MUC5AC. The MUC5B expression is influenced by hormones; its production peaks at midcycle, and this peak coincides with the change in mucus characteristics [31,39,40,41]. The current understanding of GFM expression during the ovarian cycle in relation to hormonal changes is summarized in Figure 3. We note that the data presented in Figure 3 were acquired mainly in small cohorts and case reports, and some from very old studies. This important topic deserves to be investigated more thoroughly using recent molecular tools. Gel-forming mucin modifications during pregnancy have not been examined yet.

### 2.3. Functions

Cervicovaginal mucus has multiple important functions. It protects the vaginal epithelium from repetitive friction during sexual intercourse, helps to ensures fertility [42], and stops or selectively restricts sperm transport within the female reproductive tract only during the ovulation phase through modulation of the size of the pores in the GFM network [30,32,43]. Cervicovaginal mucus also prevents colonization of the vaginal cavity by unwanted microorganisms. The vaginal epithelium, CVM, and the vaginal protective flora are intimately linked to ensure a protective role in the host. Housing, protection, and nutrients necessary for proper maintenance of the commensal flora are all provided by the mucus gel in the vaginal cavity. This host–vaginal microbiota mutualism (positive interactions between species) participates in the formation of the vaginal mucosal barrier against infectious agents such as bacteria, viruses, fungi, and protozoa [27]. The mucosal barrier prevents direct contact of microorganisms with the vaginal epithelium.

The GFMs network has an average pore size of approximately 350 nm as shown by the observations that the effective diffusivity of nanoparticles and particles larger than the size of the CVM mesh can be trapped by steric hindrance, whereas biochemical interactions with proteins and molecules can selectively modulate mucus permeability [44,45,46,47]. The interactions between mucus and the bacterial lipids and surface proteins help to slow down pathogen diffusion and help to ensure a stronger immune system response [44,48]. Foreign particles trapped in CVM can also be ejected mechanically from the WGT during CVM renewal and/or eliminated by the release of many immune cells during menstruation [49,50]. Thus, CVM helps fertility by countering vaginal infections, which can lead to stillbirths and preterm labor [51].

## 3. The Cervical Mucus Plug

**Figure 4:**
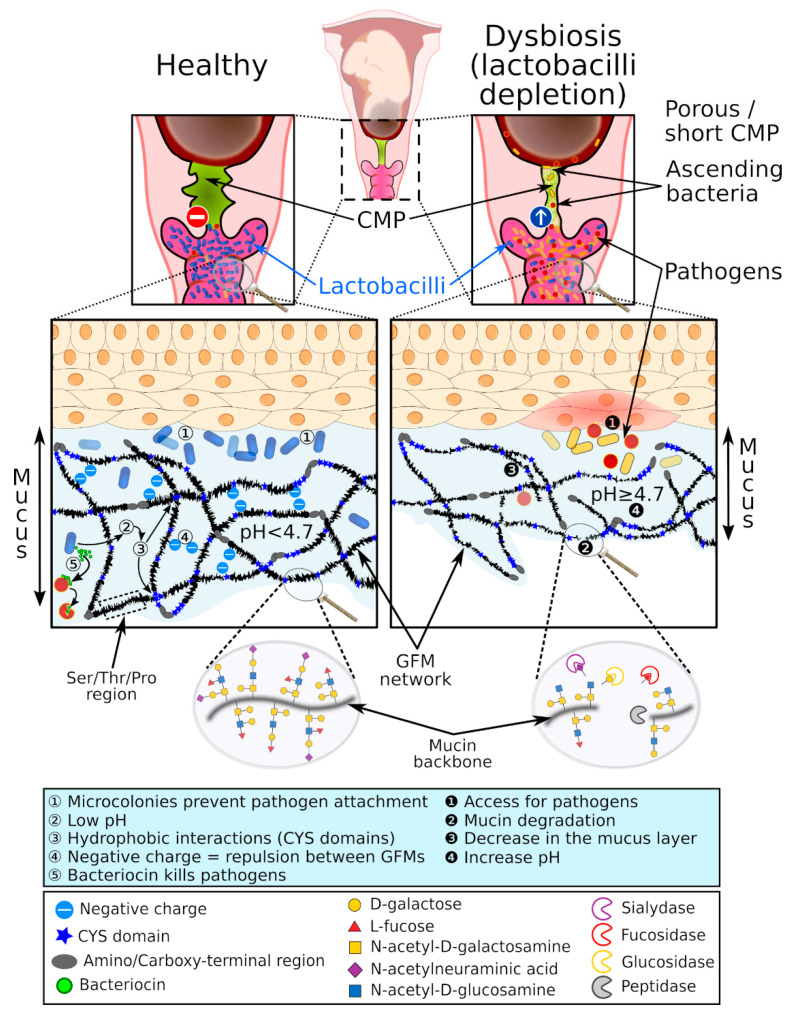
Bacterial vaginosis in the pregnant woman predisposes to ascending bacterial infection. During a healthy pregnancy, women produce a dense cervical mucus plug (green) that prevents vaginal flora from reaching the uterine cavity (left). During vaginal dysbiosis (right), the depletion of lactobacilli (blue) promotes the proliferation of pathogens in the vagina (red and yellow), which can more easily reach the uterine compartment if the cervical mucus plug (CMP) is smaller and/or too porous.

During pregnancy, the cervical epithelium produces a large and dense mucoid structure, known as the CMP. The mucus of the CMP continues to flow into the vaginal compartment throughout the pregnancy so that the CMP always contains freshly synthesized mucus. Abnormalities of the CMP, such as short length and excessive porosity, are associated with uterine infection and preterm birth [52,53]. Animal and human studies indicate that intra-amniotic infection through the cervical route triggers an inflammatory response leading to preterm labor [54]. The CMP fills the full cervical canal, obstructs the entrance into the uterine compartment during pregnancy, and is ejected just before delivery [6]. Its main role seems to be to seal the uterus to prevent vaginal flora ascension toward the uterus, which must remain sterile (Figure 4). Only a few beneficial bacteria have been found in the CMP fraction close to the vagina [55]. The CMP is mainly composed of mucus and antimicrobial compounds.

To date, the CMP has been described in only domestic mammals, including mares, cows, ewes, and monkeys [56,57,58,59] and is suspected to occur in dogs, guinea pigs, mice, and rats [60,61,62,63]. It is not surprising that the GFM composition of the CMP is even less known. The GFMs, MUC2, MUC5AC, and MUC5B, are three GFMs detected by Western blotting; MUC2 is found in very small amounts, whereas MUC5AC seems to be highly expressed [11]. The viscoelasticity of CMP is increased by high TFF3 secretion; the reported median concentrations of TFF1, TFF2, and TFF3 were reported as 3.1, 1.1, and 1000 nmol/g, respectively, in a study of spontaneously shed CMPs from 14 women in active labor [64].

This physical GFM network barrier is also lined with a chemical barrier. It is believed that the CMP represents a biological reservoir for antibiotics, which are cationic molecules. This explains the high content of the AMPs found in the CMP, including secretory leukocyte protease inhibitor, elastase, lysozyme, neutrophilic peptides 1–3 and β-defensin 1, cathelicidin, elafin, and lactoferrin [28,65,66,67]. Large numbers of immune cells, such as phagocytes, neutrophils, and macrophages, are also entrapped in the CMP [55,68]. Immunoglobulins, mostly IgG and IgA, and, to a lesser extent IgM, are secreted into the CMP [68].

## 4. The Vaginal Microbiota

### 4.1. Vaginal Lactobacilli

The vaginal cavity is colonized by bacteria, fungi, viruses, and even protozoa. The alpha diversity among all human microbiomes is the lowest in the vagina, where there is a predominance of *Lactobacillus* spp. [69]. It seems that Lactobacillus-predominant vaginal flora in women is unique among the mammalian kingdom [70]. Six types of vaginal microbiota, called community state types (CSTs), have been described [71,72] (Table 2). Community state type I is dominated by *L. crispatus*, CST II by *L. gasseri*, CST III by *L. iners*, CST IV-A and CST IV-B by different taxa composed by anaerobic bacteria, and CST V by *L. jensenii* [73]. The vaginal bacterial community differs according to ethnic origin. Community state type I is predominant among Caucasian and Asian women, and CST IV is the major form among Black women [72,74]. Community state types I and III are more common in pregnant women without complications who deliver at term than in nonpregnant women [73]. *Lactobacillus iners’* dominance and high diversity of vaginal microbiota are associated with increased risk of preterm birth [74].

The beneficial effect of lactobacilli in the WGT is well documented except for *L. iners*, which is mainly associated with vaginal dysbiosis. Lactobacilli can colonize the whole vaginal cavity because of their bacterial surface proteins, such as fimbriae, and their adhesion to the surface of the vaginal epithelium prevents pathogen adhesion [77,78]. Lactobacilli are fed by secretions of the WGT such as by-products of glycogen breakdown by amylase and mucin carbohydrates. In turn, vaginal lactobacilli secrete molecules with beneficial effects on the genital tract [79].

Lactobacilli are among the lactic acid bacteria (LAB) that produce lactic acid. The presence of lactic acid decreases the pH in the vagina and contributes to the acidic environment that strengthens the antimicrobial properties against other microorganisms, which emphasizes the strong mutualistic relationships between the host and commensal vaginal flora [55,80] (Figure 4). The acidic environment prevents the proliferation of sensitive pathogens but only has a small effect on tolerant lactic acid microorganisms [81]. Virucidal activity of lactic acid has been observed against HIV and the herpes simplex virus [82,83]. At physiological concentrations, lactic acid appears to also exert an immunomodulatory effect through a mechanism that is independent of the pH [84].

To maintain their optimal physiological state in the vagina, lactobacilli have developed multiple mechanisms to protect their ecological niche from opportunistic pathogens. It is well known that lactobacilli secrete antimicrobial molecules such as bacteriocins. Furthermore, production of hydrogen peroxide (H_2_O_2_) by lactobacilli in vagina has been noted, but any antibiotic proprieties at the physiological concentration of H_2_O_2_ remain to be shown [78,85,86,87]. Coaggregation of lactobacilli with certain pathogens represents another defense mechanism by preventing the binding of pathogens adhesins or receptors to host receptors or ligands. This topic has been reviewed by Kovachev [78]. The increased interest in LAB for vaginal health has led to new treatments for dysbiosis. For example, the two LAB, *L. plantarum* and *L. crispatus*, whose presence is associated with healthy vaginal flora, are used as probiotics for the prevention of recurrent bacterial vaginosis [88,89]. The other common strategy is to stimulate the growth of endogenous probiotics by administering prebiotics such as fructo-oligosaccharides [90].

### 4.2. LAB Interactions within CVM

The antimicrobial properties of LAB in the vagina are important for protecting the host from sexually transmitted infections, but a Lactobacillus-dominated vaginal flora is not sufficient to protect the WGT fully. Cervicovaginal mucus, vaginal lactobacilli, and host cells are highly connected, which ensures the best protection and maintains the mucosal barrier homeostasis to protect the host from external pathogens [91,92]. Several mechanisms ensure this connection between the three partners within CVM, including acidification by lactic acid, production of reactive oxygen species, direct contact between secreted mucins and cells, and diffusion of signaling molecules. Acidification of CVM by LAB induces modification of the GFM network by exposing the hydrophobic domains of mucins [93]. Such modification was suggested to improve HIV trapping [94]. It is also possible that H_2_O_2_-derived free radicals produced by lactobacilli increase mucin cross-links to stiffen the mucus gel as reported in the airway mucus for patients with cystic fibrosis [95]. Another defense mechanism is the ability of vaginal LAB to ensure a high adhesion capacity within the mucosal barrier, which limits the number of sites available for pathogen attachment by producing cell surface proteins that adhere to and build a biofilm on the mucosal surface [96,97]. Several adhesins have been identified to promote adherence of vaginal lactobacilli to stratified squamous epithelial cells [98,99]. Whether LAB bind to GFMs in the WGT remains unclear, although this is well documented in the intestine [100].

## 5. Bacterial Vaginal Infection

The three different types of relationships between the host and the microbiota are (1) commensalism (i.e., benefits for one organism but no effects on the other), (2) mutualism (i.e., benefits for both organisms), and (3) parasitism (i.e., benefit for one organism to the detriment of the other). The relationships between flora and host can evolve, as opportunistic microorganisms can promote the onset of disease in certain circumstances such as in the context of vaginal dysbiosis.

### 5.1. Vaginal Dysbiosis and Bacterial Vaginosis

Bacterial vaginosis is a common bacterial vaginal dysbiosis and is characterized by vaginal depletion of lactobacilli. The decrease in protective lactobacilli promotes proliferation of opportunistic pathogens, which increases the susceptibility to symptomatic or asymptomatic infections (Figure 4). The prevalence of vaginal dysbiosis is 15% to 50% depending on ethnic origin, socioeconomic level, and sexual practice, and seems to be higher in smokers [5,101]. Several factors are associated with the onset of vaginal dysbiosis, such as a lack of hygiene, use of lubricants or shared vaginal toys, unprotected sexual activity, menopause, and frequent use of antibiotics that alter the normal urogenital flora and predispose to colonization by uropathogenic bacteria [102,103,104,105]. Vaginal dysbiosis can be evaluated using the Nugent score, which is based on the bacterial morphology of vaginal bacteria, or by using a molecular methodology to determine the composition of the vaginal flora [106,107]. However, validation of vaginal dysbiosis can be tedious for women with the CST III (*L. iners*) or CST IV (anaerobic bacteria).

Bacterial vaginosis is one of the most common bacterial vaginal infections in women of childbearing age [108]. Bacterial vaginosis is associated with vaginal and uterine infection, preterm labor, pelvic inflammatory disease, and acquisition and transmission of sexually transmitted disease [109]. In 1983, Amsel et al. [110] described the symptoms of bacterial vaginosis as an unpleasant vaginal odor associated with increased biogenic amines in the vagina, a viscous gray discharge, increased vaginal pH, and the presence of exfoliated epithelial cells with adherent bacteria clue cells. The formation of a polymicrobial biofilm is often initiated by *Gardnerella vaginalis* that provides the scaffold for attachment of *Prevotella bivia*, *Atopobium vaginae*, and sometimes *L. iners* [111].

### 5.2. Mucosal Barrier during Bacterial Vaginosis

The properties of the CVM barrier are associated with the composition of the vaginal microbiota. For example, CVM colonized predominantly by *L. crispatus* can provide a barrier to HIV, whereas CVM in association with a dysbiotic vaginal flora exhibits reduced barrier properties [91,112]. This can be explained by three main factors. First, the vaginal microenvironment is altered during bacterial vaginosis. Vaginal depletion of lactobacilli correlates with decreased lactic acid production and increased pH (≥4.7). Combined with the absence of H_2_O_2_-producing lactobacilli [113], the dysbiotic vaginal microenvironment may interfere with the GFM network, as described above, and this can reduce the physical barrier and mucoadhesive properties of CVM [112]. Some bacteria associated with bacterial vaginosis can secrete mucinases, which are frequently detected in bacterial vaginosis [114]. Mucinases include aminopeptidases, sialidase, α- and β-galactosidase, α-fucosidase, α-glucosidase, and N-acetyl-glucosaminidase. Mucinases are mucolytic enzymes involved in the degradation of mucin carbohydrate residues and the mucin backbone leading to disruption of the interactions between mucins [114,115,116]. It has been reported that the extracellular sialidase of the vaginal pathogen *G. vaginalis* hydrolyzes and metabolizes sialic acid from mucin *O*‑glycans and sialoglycoproteins such as secreted IgA found in CVM [117]. Removal of the terminal sialic acid residue is the first step of GFM deglycosylation and exposes the underlying oligosaccharide residues to the collective action of glycosidases associated with bacterial vaginosis. The underglycosylated GFM is then exposed to bacterial aminopeptidases that degrade the mucin backbone (Figure 4).

Second, changes in CVM composition are not related solely to GFMs or other sialoglycoproteins. The large amounts of cytoskeleton and metabolic proteins detected in CVM of bacterial vaginosis-positive women are biomarkers of degradation of the epithelial cell layer [118]. During bacterial vaginosis, clue cells can be observed. Loss of epithelial integrity may be caused by endotoxins secreted by pathogenic bacteria. This is illustrated by the fact that the cytolysin vaginolysin from *G. vaginalis* is a pore-forming toxin, whose concentration was reported as two-fold higher in *G. vaginalis* isolated from women with bacterial vaginosis than in the bacteria isolated from healthy women [119].

Third, a change in the profile of proteins involved in immunity is observed during bacterial vaginosis. One example is the partial or extensive degradation of IgA and IgM reported in vaginal washings of patients with bacterial vaginosis [120]. Another good example is the interaction between the recognition of pathogen-associated molecular patterns with the Toll-like receptors of the host. This interaction induces an increase in human pro-inflammatory interleukins 1 (IL-1β), IL‑8, and tumor necrosis factor (TNF) along with AMP overproduction, which can degrade the mucus layer [27]. For more information about variation in soluble immune factors, we refer the reader to the article by Campisciano et al. [121]. In relation to the role of immune cells, more CD4 T cells with the HIV coreceptor CCR5 were found in the vagina of bacterial vaginosis-positive women; the presence of these cells correlates with a higher risk of acquiring and transmitting HIV [122,123].

Together, the depletion of lactobacilli, colonization by pathogenic bacteria, and the immune response contribute to the disruption of the vaginal mucosal barrier observed in women with bacterial vaginosis. Degradation of the mucosal barrier and epithelial cell layer provides nutrients and may promote pathogenic bacterial adhesion and colonization in the vagina. In addition, pathogens associated with bacterial vaginosis can locally disrupt CVM immunity by modulating the production of host cytokines and AMPs [124,125].

### 5.3. Bacterial Vaginal Infection during Pregnancy

Bacterial vaginosis is an important public health issue because it increases the risk of uterine infection and intrauterine infection, which seems to account for 25–40% of preterm births [126]. Preterm birth is a global health problem; 15 million babies are born too early (<37 weeks of gestation). Several factors have been identified, such as advanced maternal age [127], but many spontaneous preterm births occur in women with unknown risk factors. The mechanisms by which bacterial vaginosis can lead to preterm birth remain unclear, but the host response to pathogens in the vagina and uterus is considered to be the main cause. Microorganisms identified in the amniotic fluid are generally similar to those found in the vagina, which suggests that the cervical canal is the most common pathway for bacteria to enter the uterine cavity [128]. As discussed above, the CMP plays an important role in preventing the ascent of infection, and a shorter, more permeable, and less mucoadhesive CMP has been found in women at high risk for preterm birth compared with those at low risk [53].

It seems that an altered CMP allows the dysbiotic vaginal flora to reach the uterine cavity (Figure 4). Gel-forming mucins are mainly responsible for the viscoelastic properties of the CMP and altered permeability properties of the CMP likely result from underproduction or degradation of GFMs and/or the modification of mucin-associated glycans by bacteria usually associated with a dysbiotic vaginal flora, as discussed above. It has also been suggested that a decrease in sialic acid residues in GFM carbohydrates might increase the net charge of mucins, and thereby alter the mucoadhesive properties of the CMP and repulse cationic AMPs [53]. This could explain the insufficient antimicrobial properties of the CMP for killing some pathogenic bacteria such as Group B *Streptococcus* and *Ureaplasma parvum* [55,67].

Overproduction of pro-inflammatory mediators may be responsible for preterm labor or premature rupture of the membranes. The few studies that have investigated the link between bacterial vaginosis, pro-inflammatory mediators, and preterm birth showed that bacterial vaginosis induces a cytokine response that is twice as high in pregnant women as in nonpregnant bacterial vaginosis-positive women. In that study, the vaginal concentration of IL-1β correlated with sialidase and prolidase levels, which highlights the flexibility of innate defense [129,130,131]. Moreover, prostaglandin, IL-1⍺, IL-6, IL-8, and TNF-⍺ concentrations were significantly higher in the CVM of pregnant women with bacterial vaginosis than in healthy pregnant women [129,132,133].

Two common strategies are used against bacterial vaginosis. The most common is the use of antibiotics, usually metronidazole and clindamycin, which are administered orally or vaginally to eliminate strictly unwanted anaerobic bacteria that proliferate in bacterial vaginosis [134]. However, antibiotic treatment has limitations because the recurrence rate is estimated at 50% after 6–12 months and the risk of side effects such as preterm labor, premature rupture of the membranes, metallic taste, nausea, dizziness, vomiting, heartburn, headache, skin rash, or diarrhea [134,135,136]. The second strategy is to restore the dysbiotic vaginal flora with beneficial Lactobacillus by the administration of probiotics or prebiotics. However, relapses often appear a few weeks or months after treatment is stopped [136,137]. Emerging therapeutic strategies against bacterial vaginosis have been proposed including treatment with plant-derived compounds, natural AMPs (bacteriocins), or acidifying agents (lactic acid, vitamin C) [136]. However, to date, there is no satisfactory therapy. Thus, the CVM and the CMP appear relevant to the development of alternative therapeutic strategies, but more studies of their composition and properties are required.

Studies of the CMP in humans are limited for obvious ethical reasons because of the risk to both the mother and fetus. Nonhuman alternatives attempt to reproduce the complex environment of CVM and the CMP. By allowing the use of large samples, including control subjects, needed for valid statistical analysis, animal models remain critical to the study of the relationship between CMP properties and preterm birth.

## 6. Mouse Models for the Study of CVM and the CMP

The advent of biotechnologies to modify mammalian genomes and gene invalidation has led to a better understanding of gene function. The laboratory mouse model remains the most common model used in research to examine bacteria–host interactions, because the mouse shares many genetic, physiological, anatomical, and metabolic characteristics with humans, is cheaper than using larger mammals, and is easy to manipulate in large numbers. Mouse GFMs are now well characterized, and many tools are available, such as specific antibodies and GFM-knockout mice, whereas mucins are poorly characterized in other mammalian laboratory models. In this final section of the review, we discuss the mouse as a valuable model for studying CVM and learning more about preventing bacterial vaginosis and GFMs in the mouse genital tract (MGT).

### 6.1. Anatomy and Physiology of the MGT

As in the WGT, the MGT includes a lower genital tract (vulva, vagina, and cervix) and an upper genital tract (uterus, oviducts, and ovaries) (Figure 1). The vagina is muscular and extends from the vulva to the cervix. The mouse has a keratinized vaginal epithelium, whereas women have a nonkeratinized squamous vaginal epithelium. In both the human and mouse, the ectocervical canal is lined by a stratified squamous epithelium that is continuous with the vaginal epithelium. The mouse has a bicornuate uterus, which means the corpus is separated into two lateral horns, whereas women have a pear-shaped uterus. Both the human and mouse uterus epithelium comprises simple columnar cells (for more details see Reference [138]).

As in the WGT, the MGT is under the continuous control of progesterone and estradiol. The estrus cycle lasts approximately 4–5 days in fertile mice and can be divided into four stages. Proestrus corresponds to the preovulatory phase in the human. Ovulation occurs during the estrus phase, and metestrus and diestrus correspond to the luteal phase in the human [139]. The estrous cycle can be interrupted by anestrus, pseudopregnancy, and pregnancy. The mouse gestation time is relatively short (18.5–21 days) and the litter size is usually 5–15 pups.

### 6.2. Vaginal Microbiota

Few publications have reported on the mouse vaginal microbiota. Bacterial abundance in the mouse vagina is lower than in the WGT according to older studies [75,140]. In contrast to the human vagina, the mouse vagina does not have predominant lactobacilli but contains more Gram-positive bacteria than Gram-negative bacteria [141,142,143]. Culture-based studies have reported *Staphyloccocus spp.*, *Enterococcus faecium*, *Escherichia coli*, *Proteus mirabilis*, *Streptococcus spp.*, and lactobacilli [142]. Changes in the composition of the vaginal flora determined by culture methods according to the estrus cycle have been observed with a higher number of bacteria during the estrus phase [75]. The number of lactobacilli ranges from 10^1.9^ CFU to 10^3.3^ CFU/vagina depending on the estrus cycle, and lactobacilli were found to be present in only 50% of mice in the estrus phase. One hypothesis is that the proliferation of bacteria during estrus is favored by an increase in mucus secretion. The mouse microbiome identified by next-generation sequencing shows variations within vaginal samples, probably related to the estrus cycle. Vaginal communities differ between the cecum and lung communities and include *Streptococcus*, *Acinetobacter*, *Sphingmonas*, *Enterococcus*, and *Polaromonas* [144]. More recently, a study that used Illumina MiSeq system reported five mouse CSTs (Table 2) and that mouse vagina houses mainly *Staphylococcus spp.* and *Enterococcus spp.*, but lactobacilli were poorly represented [76]. There is no evidence in the literature of commensal *Mollicutes* and no virus has been described in the MGT.

### 6.3. Mouse Cervicovaginal Mucus

Although the laboratory mouse is the model of choice for studying the in vivo interactions between the microflora and the host, few data have been published on the mucus of the MGT and even less is known about mouse GFMs. Little has been known until very recently about the large protein and cDNA sequences (>12 kbp) of GFMs and their complex regions because of the lack of specific antibodies and the impossibility of obtaining knockout mice for GFMs until the corresponding genes were characterized. Histological studies using Periodic acid–Schiff (PAS) staining of mouse vaginal tissues have shown abundant PAS-positive granules in the stratified vaginal epithelium, which suggests the presence of mucus cells in the vagina [145]. Among the five mouse GFMs, only Muc5b has been found in the MGT and, as in women, Muc5b appeared to be hormonally regulated and overexpressed in the proestrus stage [146,147].

### 6.4. Transgenic Mouse Models to Study CVM

Several mouse knockout models for the three GFM genes *Muc2*, *Muc5ac*, and *Muc5b*, have been developed to examine further the biological role of the encoded mucins. However, little is known currently about the GFMs in CVM and the CMP because these knockout lines have not been used to explore the GFM functions in the MGT [148,149,150,151,152]. Current knowledge about the MGT has come mainly from experiments using the reporter Muc5b-GFP mouse [146]. The GFP-reporter tag was used to observe directly the production of Muc5b in the mouse vagina, in contrast to GFMs in women, which are found mainly in cervical tissue. As discussed above, CVM and the CMP are intimately associated with vaginal health. Cervicovaginal mucus in mice has been poorly studied and the mouse CMP, if it exists, has not been examined yet. The exploration of mucus functions in the CVM and CMP is thus nascent with the very recent availability of transgenic mouse models.

Because GFMs are large multidomain macromolecules, the study of the in vivo function of each domain can be examined using transgenic mice with a specific GFM domain in the mucus gel. To date, only our transgenic lines to study the CYS domain have been obtained (Tg^(Tff3/MUC5B)208Jlcd^ and Tg^(Tff3/MUC5B-meGFP)222Jlcd^). These transgenic mice secrete with Muc2 in their intestinal mucus a molecule made of 12 consecutive CYS domains from the human MUC5B. The mucin network mesh is tighter in transgenic mice than in the control wild-type mice, which leads to a decrease of motility of spermatozoa and bacteria such as *Salmonella enterica*, and bloodstream infection by intestinal bacteria after chemotherapy [153,154,155]. If the high TFF3 expression level reported in the human CMP [64] is conserved in the mouse, we expect that our transgenic mouse, which is driven by the mouse Tff3 promoter, will help in the understanding of the function of the mucin CYS domain in the mucus properties of the CMP and its contribution to the prevention of uterine infection. Another approach for studying the CVM and CMP mucus and their microbiota interaction would be to use mice deficient in glycosyltransferases [156,157], although this genetic modification will affect GFMs as well as all molecules carrying mucin-type *O*-glycans.

### 6.5. Mouse Models of Bacterial Vaginosis

Ideally, a relevant pathogen for studying vaginal infections must induce similar symptoms and diseases in the animal model with the same route of infection as in women. Today, there is no perfect model for studying vaginal infection, but mice can be a compromise for the reasons explained above and because many microorganisms can mimic WGT infection. The mouse was first used as a model to study vaginal infections in the second part of the 20th century [158,159,160]. Since then, the number of mouse models has increased steadily, and now all types of microorganisms associated with sexually transmitted infections have been examined in mice including viruses [161,162,163,164,165], protozoa [166,167], intracellular bacteria [168,169], and fungi [170].

Among bacteria, *G. vaginalis* is one of the best pathogens for simulating bacterial vaginosis in mice [171,172]. The effects caused by this pathogen are similar to those observed in bacterial vaginosis in women, including the presence of sialidase activity and clue cells, and bacterial vaginosis increased the ascent of pathogens into the uterus [173]. Only a few studies have attempted to mimic preterm birth in mice. The first issue is to define prematurity in mice because gestation can differ between murine lineages. Mouse models of preterm birth induced by bacterial infection have been reviewed recently, and the biomarkers suggested to define premature birth include skin permeability, skin histology, lung morphometry, and gene expression [174]. Researchers usually consider 18 days of gestation time or less as preterm.

Preterm birth has been induced by different experimental manipulations and reproductive conditions that differ between laboratories. Although *U. parvum* could induce preterm birth in mice [62], *E. coli* with a specific lipopolysaccharide serotype appeared to be very effective in inducing preterm labor. For example, preterm parturition was obtained in half of the wild-type mice after vaginal exposure to 10^7^ CFU of live *E. coli* serotype O55 [175]. Waddington et al. [176] created a mouse model of preterm birth induced by a bioluminescent *E. coli* that allowed tracking bacterial infection in situ and found the reporter *E. coli* in the gastrointestinal and respiratory tracts of the fetuses. A model of ascending *U. parvum* in the uterus of pregnant mice has also been developed using experimental infection combined with mild cervical injury induced by spermicide, which led to an increase in preterm birth rates of almost 30% [62]. However, mouse models to explore the relationship between the CMP properties (length, porosity) are lacking and would be informative for extending the current knowledge about the importance of the CMP in preterm birth [53,177].

## 7. Concluding Remarks and Perspectives

Cervicovaginal mucus is essential for women’s health. It is a key player in fertility and fecundity and participates actively in vaginal flora homeostasis and in the innate immune barrier against external aggression. It protects the WGT from bacterial vaginosis and sexually transmitted infection, which, according to the World Health Organization, occur in more than one million people every day and contribute to infertility and preterm births [178,179]. Increasing antibiotic resistance jeopardizes advances in women’s health care. Consequently, new preventive or therapeutic approaches must be explored to prevent and treat vaginal infection. Mucus in CMV and the CMP may be a good candidate for developing new ways to prevent bacterial vaginosis-induced preterm birth, because it is a key component of host defense by ensuring a healthy colonized vaginal compartment and maintaining the uterine cavity free from vaginal microorganisms.

Better knowledge of GFM composition and understanding of the mucus properties of CVM and the CMP in the woman and animal models are prerequisites to developing further strategies to protect against infectious diseases. In vivo characterization of mucus is still in its infancy but will progress with the recent development of mouse transgenic lines for GFMs. However, the very small size of the mouse cervical canal and the small amount of mucus in the MGT mean that other tools that are more representative of human pathophysiology must be developed to examine CVM and the CMP mucus in larger mammals. Such studies may be more appropriate for developing vaginal drugs that can cross the mucosal barrier, which remains a major challenge [180,181].

## Figures and Tables

**Table 1:** Gel-forming mucins (GFMs) in the woman’s genital tract.

GFM		Tissue				
Name	CYS *^a^*	Bartholin’s	Cervix			Uterus
		Glands	Ecto	Endo	CMP	
MUC2 [9,10,11]	2			+	+	
MUC5AC [9,10,11]	9	+		+	+	
MUC5B [9,10,11]	7			+	+	
MUC6 [9,10]	0			+		+

*^a^* Number of CYS domains. CMP = cervical mucus plug.

**Table 2:** Community state type (CST) of the woman (10^7^–10^9^ bacteria) [71,72] and the mouse genital tract (10^3^–10⁷ bacteria) [75,76]. *L*. = *Lactobacillus,* WGT = woman’s genital tract, MGT = mouse genital tract.

CST	WGT Dominated by	MGT Dominated by
*CST I*	*L. crispatus*	*Staphylococcus*
*CST II*	*L. gasseri*	*Staphylococcus* and *Enterococcus*
*CST III*	*L. iners*	*Enterococcus* and *Lactobacillus*
*CST IV*		*Streptococcus*
*CST IV-A*	Strictly anaerobic bacteria	
*CST IV-B*	Atopobium and anaerobic	
	bacteria	
*CST V*	*L. jensenii*	Mix of bacteria

## Abbreviations

AMP	antimicrobial peptide
CMP	cervical mucus plug
CVM	cervicovaginal mucus
CFU	colony-forming unit
CST	community state type
GFM	gel-forming mucin
HIV	human immunodeficiency virus
IL	interleukin
Ig	immunoglobulin
LAB	lactic acid bacteria
MGT	mouse genital tract
PAS	Periodic acid–Schiff
TFF	trefoil factor family
TNF	tumor necrosis factor
WGT	woman’s genital tract
